# Nasal high–flow oxygen therapy in patients with hypoxic respiratory failure: effect on functional and subjective respiratory parameters compared to conventional oxygen therapy and non-invasive ventilation (NIV)

**DOI:** 10.1186/1471-2253-14-66

**Published:** 2014-08-07

**Authors:** Norbert Schwabbauer, Björn Berg, Gunnar Blumenstock, Michael Haap, Jürgen Hetzel, Reimer Riessen

**Affiliations:** 1 Department of Internal Medicine, Medical Intensive Care Unit, University of Tübingen, Otfried-Müller-Str, 10, Tübingen 72076, Germany; 2 Department of Clinical Epidemiology and Applied Biometry, University of Tübingen, Tübingen, Germany; 3 Department of Internal Medicine, Division of Pulmonary Medicine, University of Tübingen, Tübingen, Germany

**Keywords:** Respiratory failure, Oxygen, High-flow nasal cannula, Non-invasive ventilation, Venturi mask

## Abstract

**Background:**

Aim of the study was to compare the short-term effects of oxygen therapy via a high-flow nasal cannula (HFNC) on functional and subjective respiratory parameters in patients with acute hypoxic respiratory failure in comparison to non-invasive ventilation (NIV) and standard treatment via a Venturi mask.

**Methods:**

Fourteen patients with acute hypoxic respiratory failure were treated with HFNC (FiO_2_ 0.6, gas flow 55 l/min), NIV (FiO_2_ 0.6, PEEP 5 cm H_2_O Hg, tidal volume 6–8 ml/kg ideal body weight,) and Venturi mask (FiO_2_ 0.6, oxygen flow 15 l/min,) in a randomized order for 30 min each. Data collection included objective respiratory and circulatory parameters as well as a subjective rating of dyspnea and discomfort by the patients on a 10-point scale. In a final interview, all three methods were comparatively evaluated by each patient using a scale from 1 (=very good) to 6 (=failed) and the patients were asked to choose one method for further treatment.

**Results:**

PaO_2_ was highest under NIV (129 ± 38 mmHg) compared to HFNC (101 ± 34 mmHg, p <0.01 vs. NIV) and VM (85 ± 21 mmHg, p <0.001 vs. NIV, p <0.01 vs. HFNC, ANOVA). All other functional parameters showed no relevant differences. In contrast, dyspnea was significantly better using a HFNC (2.9 ± 2.1, 10-point Borg scale) compared to NIV (5.0 ± 3.3, p <0.05), whereas dyspnea rating under HFNC and VM (3.3 ± 2.3) was not significantly different. A similar pattern was found when patients rated their overall discomfort on the 10 point scale: HFNC 2.7 ± 1.8, VM 3.1 ± 2.8 (ns vs. HFNC), NIV 5.4 ± 3.1 (p <0.05 vs. HFNC). In the final evaluation patients gave the best ratings to HFNC 2.3 ± 1.4, followed by VM 3.2 ± 1.7 (ns vs. HFNC) and NIV 4.5 ± 1.7 (p <0.01 vs. HFNC and p <0.05 vs. VM). For further treatment 10 patients chose HFNC, three VM and one NIV.

**Conclusions:**

In hypoxic respiratory failure HFNC offers a good balance between oxygenation and comfort compared to NIV and Venturi mask and seems to be well tolerated by patients.

**Trial registration:**

German clinical trials register: DRKS00005132.

## Background

Acute respiratory failure can be functionally classified as hypoxic type 1 and hypercapnic type 2 respiratory failure. Hypercapnic respiratory failure is a clear indication for non-invasive or invasive ventilatory support, whereas the administration of oxygen is the primary treatment in mild and moderate hypoxic respiratory failure [1,2]. With additional oxygen supply, the fraction of oxygen in inspired air (FiO_2_) is raised above that of the normal atmosphere to avoid or correct hypoxemia with subsequent tissue hypoxia [3].

Oxygen applicators can be basically divided into low-flow and high-flow devices [4]. Low-flow devices such as nasal catheters, cannulas or simple masks deliver a limited flow of 100% -oxygen (1 – 15 l/min maximum) which is mixed with the total inspiratory gas flow of the patient. The effective inspiratory oxygen concentration (EIO_2_) depends on the respiratory flow and breathing pattern of each individual patient and therefore is difficult to estimate in advance [5].

Modern high-flow nasal cannula (HFNC) devices equipped with an active humidification chamber can provide gas flow rates up to 70 l/min, which are higher than the patient’s respiratory flow, and therefore allow a controlled delivery of a defined FiO_2_ up to 1.0, independent of the breathing pattern of each patient [6]. HFNC might also reduce the work of breathing [7]. HFNC requires active heating and humidification of the respiratory gases in order to prevent damage of the respiratory epithelium and reduces discomfort during therapy [8,9]. Lastly, HFNC is increasingly used in intensive care and emergency medicine [10,11].

Oxygen can also be applied via nasal, facial or full-face masks using non-invasive ventilation (NIV). NIV has the advantage that the use of positive end-expiratory pressure (PEEP) can induce alveolar recruitment and improve hypoxemia by reducing shunts. Despite these theoretical advantages, the use of NIV in hypoxemic respiratory failure is much less established than it is in hypercapnic respiratory failure [12-14].

In this study we compared oxygen therapy with HFNC to NIV and Venturi mask in patients with acute hypoxic respiratory failure. We used a short-term experimental protocol with a focus on respiratory and circulatory parameters in combination with subjective ratings by the patients. We specifically tried to evaluate HFNC in terms of patient comfort and relief of dyspnea in comparison to established standard methods.

## Methods

### Patients

Patients with primary hypoxic respiratory failure (PaO_2_ < 55 mmHg breathing room air) of acute onset admitted to the Medical ICU were enrolled in the present study from March 2009 to March 2011. Patients were screened for participation in the study during morning rounds and during the day shift and received a standard work-up for respiratory failure including a chest x-ray. The PaO_2_ of <55 mmHg breathing room air was chosen as a compromise between the severity of respiratory failure and patient safety during the study protocol. Exclusion criteria were defined as follows: clinical evidence for cardiac pulmonary edema (history, clinical examination, chest x-ray, echocardiography), COPD and/or ventilatory failure (history, clinical examination, chest x-ray, PaCO2 > 50 mmHg), hemodynamic instability, contraindications to NIV, impaired consciousness or disorientation, and inability to give informed consent. The Ethics Committee of the University of Tübingen approved the protocol. All subjects gave informed written consent and underwent the standard procedures of the protocol, including medical history, review of medications, physical examination and routine blood tests.

### Study protocol

**Figure 1:**
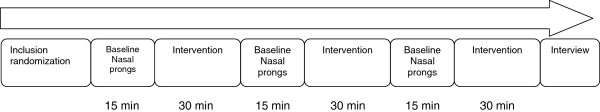
**Flowchart.** During the intervention patients were treated in a randomized order with either high-flow nasal cannula (HFNC), Venturi-mask (VM) or with none - invasive ventilation (NIV).

The order of the experimental protocol was randomly assigned (Figure 1). The assignment of patients to the sequence in which the three oxygen applicators were applied was randomized. Each intervention was preceded by a 15-minute baseline period, in which oxygen was administered via conventional nasal prongs. The gas flow was set to achieve a peripheral oxygen saturation (SpO_2_) of ≥ 88%. This gas flow was used in all subsequent baseline phases. The interventions themselves had a duration of 30 min. The patients were allowed to stop an intervention prematurely (e.g. if they did not tolerate an oxygen applicator).

### Measurements

After each baseline and intervention phase, the following parameters were measured: arterial blood gases, heart rate, blood pressure, respiratory rate, and peripheral oxygen saturation. All data were documented in the electronic Patient Data Management System (PDMS, CareVue®, Philips).

In addition, after each intervention patients were asked to grade the severity of their dyspnea on a modified Borg category ratio 10 (CR10) scale. Low numbers indicated little dyspnea, high numbers severe dyspnea. A similar 10-point numeric rating scale (NRS) was used to grade general discomfort related to the oxygen applicator with low numbers indicating low discomfort. After the third intervention, the patients were asked for a global rating of all three applicators using a six-point rating system (1 = very good to 6 = failed). The patients could also choose one oxygen applicator for further treatment.

The primary endpoint was the PaO2. Secondary endpoints were respiratory rate, dyspnea (Borg-scale), discomfort (10-point NRS), PaCO2, heart rate, blood pressure, SpO_2_, global rating, and patient preference.

### Oxygen applicators used

Standard nasal prongs (AsidBonz, Herrenberg, Germany) with an oxygen flow of 4–12 L/min. were used for the baseline period (Table 1). For conventional controlled oxygen administration, patients received a Venturi mask (Unomedical, Birkerød, Denmark) with an oxygen flow of 15 L/min and a FiO_2_ of 0.6. Both oxygen applicators were used together with a closed sterile water system for conventional bubble humidification at room temperature (Respiflo, Covidien, Neustadt/Donau, Germany).

**Table 1:** Patients characteristics

**No**	**Diagnosis**	**Age**	**BMI [kg/m**^**2**^**]**	**SAPS II**	**Baseline O**_**2**_**-flow**	**Measurement in days after ICU-admission**	**Mech. venti. during ICU stay (no/after the study)**	**Hospital survival**
		**[years]**			**[L/min]**			
1	Idiopathic pneumonia syndrome	29	27	56	10	0	1 day, after	No
2	Pneumonia (respiratory syncytial virus)	69	34	55	7	6	no	Yes
3	Pneumonia (Strep. pneumoniae)	82	21	29	8	10	no	Yes
4	Pneumonia	72	30	36	6	1	no	Yes
5	Pneumonia (Staph. aureus)	78	30	48	12	15	no	Yes
6	Pneumonia, pleural mesothelioma	71	26	54	4	1	no	Yes
7	Pneumonia	76	28	55	6	0	no	Yes
8	Pneumonia	50	25	45	6	1	no	Yes
9	Adenocarcinoma lung	59	19	29	7	7	11 days, after	No
10	Respiratory failure, myelodysplastic syndrome	68	30	43	5	6	no	Yes
11	Connective tissue disease	38	29	15	8	0	no	Yes
12	Pneumonia	28	29	32	5	1	no	Yes
13	Pneumonia (influenza H_1_N_1)_	45	22	37	4	2	no	Yes
14	Alveolar hemorrhage	18	24	43	5	4	no	Yes

*BMI* body mass index, *SAPS II* Simplified Acute Physiology Score II.

High - flow oxygen therapy via a HFNC (OptiFlow®, Fisher & Paykel Healthcare Welzheim, Germany) was administered with a FiO_2_ 0.6 and a gas flow of 55 L/min using an active respiratory gas humidifier (MR 850, setting “invasive ventilation”, Fisher & Paykel Healthcare, Welzheim, Germany).

For NIV, intensive care ventilators in the pressure support mode were used according to the current guideline for NIV in respiratory failure [14]. In all patients FiO_2_ was set to 0.6 and PEEP to 5 cm H_2_O. Five patients were treated with a ventilator equipped specific NIV-mode (Servo i, Maquet, Rastatt, Germany), 9 patients were treated with a ventilator without a NIV-mode (Dräger Evita 4, Dräger, Lübeck, Germany). The pressure support above PEEP was adjusted individually to achieve a tidal volume of 6–8 ml/kg ideal body weight. The interface between the patient and the ventilator was a commercially available mouth/nose mask (UltraMirage NV, Resmed Martinsried, Germany). The ventilators were also equipped with an active respiratory gas humidifier (MR 850, setting NIV, Fisher & Paykel Healthcare Welzheim, Germany).

### Statistics

Data are presented as means ± standard deviations. Repeated measures ANOVA was used to assess the measurements taken under the three different conditions (VM, HFNC, NIV). Statistical significance was set at p < 0.05. Post-hoc pair wise comparisons using the paired t test were conducted only in the case of an overall significant ANOVA result. A sample size of 14 patients had a priori been estimated to have 80% power to detect a clinically relevant difference of 10 mmHg in PaO2 across the 3 conditions given an SD of 35 mm Hg at each level and a between level correlation of 0.80. Likewise, for both the modified Borg CR10 dyspnea scale and the patient discomfort 10-point numeric rating scale, a relevant difference of 1 scale point across the conditions given an SD of 2.5 points at each level and an between level correlation of 0.60 would be detected with 80% power, when the sample size was 14. All analyses were performed with the JMP® 9.0 statistical software (SAS Institute, Cary, NC).

## Results

Fourteen patients with hypoxic type 1 respiratory failure were enrolled in the study (Table 1). In three patients the NIV intervention phase had to be stopped prematurely because the patients did not tolerate the NIV mask. We did not observe any evidence for patient-ventilator asynchronicity during the NIV intervention phase. In one case, the blood gas analysis at the end of the NIV intervention phase could not be collected in time due to a technical problem. Measurements during the three baseline periods did not show any significant differences (Table 2).

**Table 2:** Measured variables

	**Bl 1**	**Bl 2**	**Bl 3**	**VM**	**HFNC**	**NIV**	**p-value**
HR [1/min]	90 ± 23	90 ± 23	89 ± 20	87 ± 21	89 ± 23	86 ± 21	ns
BP_mean_ [mmHg]	88 ± 16	90 ± 15	86 ± 18	88 ± 13	90 ± 16	91 ± 18	ns
RR [1/min]	28 ± 9	28 ± 9	26 ± 7	28 ± 8*	26 ± 7	24 ± 9*	*VM – NIV
							p < 0,01
SpO_2_ [%]	93 ± 3	92 ± 6	93 ± 3	95 ± 4	96 ± 3	98 ± 3	ns
pH	7.47 ± 0.06	7.46 ± 0.05	7.46 ± 0.07	7.46 ± 0.06	7.46 ± 0.07	7.44 ± 0.08	ns
PaO2 [mmHg]	67 ± 15	64 ± 10	66 ± 10	85 ± 22^#^	101 ± 34*^†#^	129 ± 38^†^*	^#^VM-HFNC
							p < 0,01
							^†^HFNC-NIV
							p < 0,01
							*VM-NIV-
							p < 0,001
PaCO2 [mmHg]	36 ± 5	38 ± 5	37 ± 5	37 ± 6	37 ± 5	39 ± 7	ns
Borg scale	3.6 ± 2.5	4.3 ± 3.2	3.9 ± 2.7	3.3 ± 2.3	2.9 ± 2.1*	5.0 ± 3.3*	*HFNC-NIV
							p < 0,05
Patient comfort	2.5 ± 1.6	4.0 ± 1.9	4.0 ± 2.3	3.1 ± 2.8	2.7 ± 1.8*	5.4 ± 3.1*	*HFNC-NIV
							p < 0,05
Global rating	n. a.	n. a.	n. a.	3.1 ± 1.7*	2.3 ± 1.3#	4.5 ± 1.7#*	*VM-NIV
							p < 0,05
							^#^HFNC-NIV
							p < 0,01

Values are presented as means ± standard deviation, *Bl* baseline, *VM* Venturi mask, *NIV* Non-invasive ventilation, *HFNC* nasal high-flow oxygen, *HR* heart rate, *BP*_*mean*_ mean arterial blood pressure, *RR* respiratory rate, *SpO*_*2*_ peripheral oxygen saturation, *PaO2* arterial pressure of oxygen, *PaCO2* arterial partial pressure of carbon dioxide.

PaO_2_ with NIV (129 ± 38 mmHg) was significantly higher than PaO_2_ with HFNC (101 ± 34 mmHg, p <0.01) (Table 2). In contrast to NIV and HFNC, PaO_2_ with VM was significantly lower (85 ± 21 mmHg, P <0.001 vs. NIV, p <0.01 vs. HFNC). The respiratory rate was slightly, but significantly lower under NIV vs. VM (24 ± 9 vs. 28 ± 8, p < 0,01). All other cardiorespiratory and blood gas parameters showed no relevant differences (Table 2).

Subjective rating of dyspnea expressed on a 10-point Borg scale was lowest under HFNC (2.9 ± 2.1), but not significantly different to VM (3.3 ± 2.3) (Table 2). Dyspnea with NIV, however, was rated significantly higher than with HFNC (5.0 ± 3.3, p <0.05). Similarily, patient discomfort was lowest with HFNC (2.7 ± 1.8) and VM (3.1 ± 2.8, ns vs. HFNC), and highest with NIV (5.4 ± 3.1 p <0.05 vs. HFNC).

In the final global rating, patients gave the best grades to HFNC (2.3 ± 1.3), followed by VM (3.1 ± 1.7, ns vs. HFNC) and NIV (4.5 ± 1.7, p <0.01 vs. HFNC and p <0.05 vs. VM). For further treatment, 10 patients (71%) chose HFNC (exact 95% CI: 42% to 92%), 3 chose VM, and 1 NIV.

## Discussion

In this study we investigated the effects of controlled oxygen therapy with HFNC compared to Venturi mask and NIV therapy in patients with mild to moderate acute hypoxic respiratory failure. The protocol was designed to compare the short-term effects on gas exchange and cardio-respiratory parameters. We also conducted a subjective patient evaluation of the three oxygen applications.

The PaO_2_ was the only gas exchange parameter that was significantly affected by the type of oxygen applicator despite an identical FiO_2_ (0.6). Not surprisingly, the highest PaO_2_ was observed under NIV, because here the PEEP of 5 cm H_2_0 might have led to alveolar recruitment and reduction of intrapulmonary shunt. It has been proposed that HFNC also can lead to some alveolar recruitment [15]. However, in a study by Groves HFNC with a gas flow of 60 l/min. increased airway pressures only up to 2,7 cm H_2_O while breathing with an open mouth, and 7.4 cm H_2_O with the mouth closed. This increase in airway pressure was considered negligible [16]. Nevertheless, a recent study of Riera and coworkers [17] shows an increase in end-exspiratory lung volume, messured by electrical impedance tomography (EIT), presumably due to the higher airway pressure levels.

The higher PaO_2_ observed under HFNC, compared to a Venturi mask, can be explained by the higher administered gas flows (up to 55 l/min). With a Venturi mask and an oxygen flow of 15 l/min the targeted FiO_2_ of 0.6 can only be achieved when the total gas flow is not higher than 30 L/min. In acute hypoxic respiratory failure often much higher inspiratory gas flows are generated. Especially in Venturi systems and other masks that are not tight fitting, this leads to an additional room-air admixture during inspiration resulting in a reduction of FiO_2_[6].

In our patients with mild to moderate acute hypoxic respiratory failure, all oxygen application systems were able to increase the mean PaO_2_ into the horizontal upper part of the oxygen binding curve, so that a normal mean arterial oxygen saturation (SaO_2_) of ≥ 95% could be achieved with all oxygen applicators. This might explain why we did not observe any relevant differences regarding the other measured cardiorespiratory parameters (respiratory rate, PaCO_2_) during the short observation period of just 30 minutes. A notable effect on these parameters was found in other studies [11,18-21] of more severe forms of hypoxic respiratory failure. In these studies, differences in the PaO_2_ attributable to the type of oxygen applicator affect the SaO_2_ in the steep part of the oxygen binding curve. In such patients, a higher SaO_2_ might reduce hypoxic respiratory drive. However, patients with these more severe forms of hypoxic respiratory failure were excluded from our study, because they are usually under severe distress and not able to comply with the experimental protocol.

With respect to patient comfort and satisfaction, in our study HFNC received the best subjective ratings regarding dyspnea, discomfort and general evaluation and was preferred by a large majority of the patients for further oxygen supplementation. Compared to Venturi mask, however, the differences were not statistically significant. We hypothesize that improved humidification of the respiratory gas using HFNC might have a minor positive effect on patient comfort. Chanques et al. investigated the effect of an active humidification system compared to a conventional bubble humidifier during oxygen therapy via a face mask using a gas flow of 5–11 l/min. Active humidification resulted in less symptoms of dehydration of the upper respiratory tract and significantly increased comfort for the patients [22]. Cuquemelle et al. similarily showed increased patient comfort when standard oxygen therapy via the nasal airways with no humidification was compared with HFNC with heated and humidified oxygen [8]. The positive effect of HFNC on respiratory gas humidification, however, might be offset by other factors affecting patient comfort such as an increased noise level which often is a problem at least with older HFNC systems. Other studies, however, also have shown that HFNC is well tolerated and effective in relieving dyspnea when compared to oxygen therapy via standard face masks [11,20]. In the study by Lenglet et al. patients with acute hypoxic respiratory failure were treated in the emergency department first with a non-rebreathing mask and were then switched to HFNC. HFNC was preferred by 76% of the healthcare givers.

Our study so far is the first study to compare HFNC with NIV in the setting of acute hypoxic respiratory failure, NIV received the worst subjective ratings in all categories although it led to the best improvement in oxygenation. Three patients terminated the NIV phase of the study early due to intolerance of the application. It should be noted that the adaptation phase to the NIV mask in our protocol was very short. So, it is possible that an individual optimization of the NIV parameters and a longer adaptation phase might have resulted in improved patient tolerance and comfort. However, patients with hypoxic respiratory failure often require prolonged periods of respiratory support, which many patients do not tolerate on NIV. In clinical practice we often use a combination of NIV and HFNC in patients with moderate to severe hypoxic respiratory failure in whom we are trying to avoid intubation (e.g. immunocompromised patients) [23,24]. A beneficial effect of NIV on dyspnea has also been shown in end-of-life patients with solid tumors, however, it was not compared to HFNC in this study setting [25]. Intermittent phases with HFNC helped to prevent skin damage, allow the patients to cough, eat, and communicate, and thus might increase patient compliance.

Our study was designed as a short term experimental study and therefore has several limitations. The duration of the interventions was with 30 minutes very short. Longer durations, however, would have made direct comparisons of the measurements more difficult. In addition, for patients in acute respiratory distress it was problematic to comply with a longer experimental protocol. Our study did not address questions regarding the correct indication for HFNC and its long-term effects. Several recent studies have demonstrated that HFNC can be applied safely in many patients with acute hypoxic respiratory failure including influenza H1N1 pneumonia [20,21]. Patients with severe respiratory failure, however, may still require mechanical ventilation. A recent experimental study showed that in mild lung injury spontaneous breathing improved lung recruitment. In severe lung injury, however, accelerated spontaneous ventilation induced more lung injury than lung-protective mechanical ventilation including muscle paralysis. High tidal volumes under spontaneous ventilation led to high transpulmonary pressures with significant stress and strain on the injured lung. The lack of a PEEP also promoted atelectasis and cyclic alveolar collapse. Finally, application of a high FiO_2_ up to 1.0, which is possible with HFNC, might further accelerate lung injury [26].

## Conclusion

HFNC is a well tolerated and an effective device for oxygen therapy in mild to moderate hypoxic respiratory failure and bridges the gap between conventional oxygen applicators, NIV, and invasive mechanical ventilation. The indications and contraindications for HFNC have to be further clarified in additional clinical outcome studies.

## Abbreviations

HFNC: High-flow nasal cannula; NIV: Non-invasive ventilation; VM: Venturi mask; PaO_2_: Partial pressure of oxygen in arterial blood; FiO_2_: Fraction of oxygen in inspired air; EIO_2_: Effective inspiratory oxygen concentration; PEEP: Positive end-expiratory pressure; COPD: Chronic obstructive pulmonary disease; PDMS: Patient Data Management System; ANOVA: Analysis of variance; Bl: Baseline; HR: Heart rate; BP_mean_: Mean arterial blood pressure; RR: Respiratory rate; SpO_2_: Peripheral oxygen saturation; PaO2: Arterial partial pressure of oxygen; PaCO2: Arterial partial pressure of carbon dioxide.

## Competing interests

Fisher & Paykl provided two Optiflow-devices at no charge for the study. Otherwise the investigators received no financial support for the study and the company was not involved in data analysis and publication of the study.

## Authors’ contributions

NS. Conception and design of the study, data acquisition, data analysis, manuscript drafting BB. Conception and design of the study, data acquisition, data analysis, manuscript editing GB. Conception and design of the study, data analysis, statistics, manuscript editing MH. Recruitement of subjects for the study, data acquisition, manuscript drafting, manuscript editing JH. Conception and design of the study, data analysis, manuscript editing RR. Conception and design of the study, recruitement of subjects, data analysis, manuscript editing. All authors read and approved the final manuscript.

## Pre-publication history

The pre-publication history for this paper can be accessed here:

http://www.biomedcentral.com/1471-2253/14/66/prepub
